# Alginate/Banana Waste Beads Supported Metal Nanoparticles for Efficient Water Remediation

**DOI:** 10.3390/polym13234054

**Published:** 2021-11-23

**Authors:** Taghreed M. Fagieh, Esraa M. Bakhsh, Sher Bahadar Khan, Kalsoom Akhtar, Abdullah M. Asiri

**Affiliations:** 1 Department of Chemistry, Faculty of Science, King Abdulaziz University, Jeddah 21589, Saudi Arabia; tfagieh@kau.edu.sa (T.M.F.); sbkhan@kau.edu.sa (S.B.K.); kaskhan@kau.edu.sa (K.A.); aasiri2@kau.edu.sa (A.M.A.); 2 Center of Excellence for Advanced Materials, King Abdulaziz University, Jeddah 21589, Saudi Arabia

**Keywords:** banana waste, alginate, beads, metal nanoparticles, water pollutants

## Abstract

Water pollution is considered a perilous issue that requires an immediate solution. This is largely because of the strong correlation between the global population increase and the amount of waste produced (most notably food waste). This project prompts the conversion of food waste into useful materials that can be used with sodium alginate as a catalytic support for metal nanoparticles. Sodium alginate/banana peel (Alg/BP) beads were prepared simply using an eco-friendly method. The prepared materials were modified using nanostructured materials to enhance their characteristics. Alg/BP beads were employed as adsorbents for metals that were then treated with sodium borohydride to produce MNPs@Alg/BP. Different MNPs@Alg/BP (MNPs = Ag, Ni, Co, Fe, and Cu) were used as catalysts for reducing 4-nitrophenol (4-NP) by NaBH_4_ to evaluate each catalyst performance in a model reaction. The results exhibited that Cu@Alg/BP was most efficient toward complete transformation of 4-NP. Therefore, Cu@Alg/BP was also used as a catalyst for the reduction of potassium ferricyanide, congo red, methyl orange (MO), and methylene blue. It was found that Cu@Alg/BP beads catalytically reduced up to 95–99% of above pollutants within a few minutes. Cu@Alg/BP beads were more selective in reducing MO among the pollutants. The catalytic activity of Cu@Alg/BP was examined by evaluating the impact of numerous parameters on MO reduction. The results are expected to provide a new strategy for the removal of inorganic and organic water contaminants based on efficient and low-cost catalysts.

## 1. Introduction

For last few decades, drinking water has been in high demand because of the rapid increase in population growth. Due to the recent expansion of civilization, several industrial products are widely used, such as plastics and phenolic products, which decompose into toxic chemical compounds that pollute the environment to a great extent. Moreover, human life and health, aquatic habitats, and plant species are significantly impacted. The existing source of pristine water is continuously contaminated by these industries and their toxic products. For example, groundwater is considered as a primary source of potable water for many countries; however, its quality is constantly deteriorating owing to the use of dyes, heavy metals from industries and fertilizers and pesticides from agricultural processes. Most rivers and ponds worldwide are remarkably polluted by the continuous discharge of various industrial effluents resulting in a remarkable negative influence on human health and the natural ecosystem. It has been reported that some highly toxic chemical species have been found in drinking water at alarming levels. Their effects are obvious through the presence of certain diseases that noticeably spread across the world such as liver disorder, kidney failure, and cancer. Therefore, environmentally friendly and cheaper adsorbent materials are urgently needed to improve the quality of water by eliminating chemical toxicants from potable water [1,2].

Numerous chemical techniques have been developed to minimize water pollution. According to the literature, various approaches have been employed to reduce or eliminate harmful pollutants from aquatic solutions such as solvent extraction, membrane and magnetic separation, oxidation, reduction, precipitation, photochemical reactions, bacterial treatment, irradiation by nuclear radiation, ion exchange, and adsorption [3]. However, some of these developed methods have their own limitations such as high operational costs and they may require highly skilled operators. The adsorption technique has an advantage compared to other methods mentioned above [4,5]; this non-destructive technique offers some benefits such as cost effectiveness and a simple operation, thus reducing the need for any high-skilled operation as well as lacking health risk since they are environmentally safe [6]. Furthermore, both soluble and insoluble contaminants can be effectively treated to provide a large adsorption capacity. Activated carbons with a large surface-area are commonly used as effective adsorbents for different pollutants [7,8].

According to the literature, many agricultural wastes have been utilized as adsorbents for various toxicants. Banana waste has been important among them due to the possibly utilization of its various parts such as fruit peels, leaves, piths, stems, and trunks [9,10]. It is worth mentioning that a wide range of contaminants have been extensively investigated using parts of banana waste as adsorbents [11]; however, many serious environmental threats can be caused by banana tree waste when it is not managed properly. Usually, banana tree waste is thrown into rivers and ponds by farmers where it slowly decomposes resulting in generation of methane and other greenhouse gases that affect the nearby ecosystem [12]. Therefore, most researchers have utilized banana waste as an efficient raw material for adsorbents against toxicants. Its effectiveness is due to its abundant availability, the existence of a large quantity of carbon compounds, a remarkable adsorption capacity against water-soluble contaminants, and inappropriate exploitation of the banana waste by farmers after fruit harvesting. Thus, the use of banana waste as efficient adsorbents is a smart and promising option for a sustainable future [13,14].

Banana fruit is considered one of the most nutritious fruit crops and it is cultivated in more than 130 countries; it has a significant economic importance since it is the most popular tropical fruit. Banana peels (BP) are an important waste produced in huge amounts owing to the considerable consumption of this fruit. The contribution of BP to the total mass of fresh fruit is about 40%. In fact, BP contains organic compounds that are rich in carbon such as pectin substances, hemicellulose, cellulose, and other low-molecular-weight compounds [9]. As mentioned above, several serious environmental issues can be caused by banana waste in particular its peel since many tons of this waste is generated daily in household garbage and fruit markets and are usually discarded in typical landfills. The natural balance of atmospheric gases is badly affected by the anaerobic digestion of banana waste biomass, which generates toxic gases [15,16]. Accordingly, banana peel should be converted into a useful material that can efficiently contribute to the development of the economy of the agricultural industry and protect the ecosystem. The utilization of banana peel waste in environmental remediation is an economically feasible solution to overcome issues that are caused by their disposal process [13].

Selection of an efficient and eco-friendly technique for environmental pollution elimination is an essential task and challenge. Nowadays, nanoparticles that are characterized by superior physical and chemical properties are one of the promising chemical compounds for environmental pollution remediation. It has been reported that many organic and inorganic environmental toxicants have been eliminated by using heterogeneous or homogenous catalysts. These effective catalysts rely on metal nanoparticles (MNPs) or metal complexes. In recent decades, heterogeneous catalysts have been preferably used owing to their valuable advantages such as the wide availability of diverse supports, high stability, and insolubility in solvents. Furthermore, recyclability/reusability is the most important benefit of such catalysts [17]. At present, tremendous attention have been paid to nanotechnology due to their wide range of environmental and other applications [18,19,20]. In fact, nanoparticles (NPs) have incomparable physical and chemical characteristics such as large surface area to volume ratios and high reactivity. Notably, many different metal nanoparticles (MNPs) can be efficiently used for environmental pollutants removal [1,2]; however, the fact that NPs usually agglomerate in a reaction mixture is a significant issue. As a solution for this issue, MNPs could be applied on the surface of solids as a catalytic support. Today, many synthetic and natural polymers can be utilized as effective catalytic supports for different kinds of heterogeneous catalysts owing to their special surfaces and good mechanical characteristics, which lead to their long-term use [1]. According to the literature, many MNPs-based polymers have been widely used for environmental applications due to their valuable advantages such as high porosity and surface area, chemical stability as well as their uniform and interconnected structures [2].

Sodium alginate is a kind of polysaccharide that is plentifully available in marine brown algae. It is naturally soluble in hot water forming a viscous solution. It is an electrostatically interactive and eco-friendly degradable compound since it contains hydroxyl (–OH) and carboxylate (–COO−) functional groups in its chemical structure [21,22]. Most importantly, various metal cations can be bound to it forming a spherical structure. However, the ionically-cross-linked hydrogel of pure alginate displays some unfavorable structural characteristics such as a weak mechanical strength and structural instability causing some limitations in its application in the field of catalysis. In order to overcome these issues, the mechanical strength could be enhanced by integrating interactable polymers or nanomaterials [23,24].

In this current study, a composite of alginate polymer with banana peels (Alg/BP) as a catalytic support for different metal nanoparticles (Cu, Ag, Ni, Co, and Fe) was successfully prepared and utilized as catalysts for the reduction reaction of some environmental pollutants, 4-NP, K_3_[Fe(CN)_6_] and three organic dyes (MO, MB, and CR), in the presence of a reducing agent (NaBH_4_).

## 2. Experimental Section

### 2.1. Materials and Method

Sodium borohydride (NaBH_4_, 97%) and Na-Alginate were purchased from BDH chemicals, UK. Organic dyes (congo red (CR), methyl orange (MO) and methylene blue (MB)), 4-nitrophenol (4-NP), and potassium hexacyanoferrate (III) were purchased from Sigma-Aldrich, USA. All other reagents were of spectral and analytical purity grade. Distilled water was used for all preparations of solutions.

### 2.2. Preparation of Alg/BP and MNPs@Alg/BP

Alg/BP nanocatalyst was prepared by dispersing BP in Alg solution. Briefly, banana peels were dried in open air and then ground into a fine powder. Then, 1.0 g of BP was blended with 50 mL of Alg and, after complete mixing, a viscous suspension was formed and poured into a syringe. AlCl_3_ (2.0 M) solution was prepared in water and used as a cross linker for preparing the hydrogel beads. The viscous Alg was kept in the syringe with mixed BP solution and added dropwise to AlCl_3_ solution. Each drop was turned into a round bead in AlCl_3_ solution due to the AlCl_3_ crosslinked Alg chains. Alg/BP beads were kept overnight in AlCl_3_ solution and then washed carefully with distilled water. The washing continued until complete removal of surplus and unreacted Al^3+^ from the Alg/BP beads. Afterwards, the Alg/BP beads were dried in open air at room temperature. The carboxyl groups of Alg cross-linked physically with cations such as Al^3+^ in the aqueous solutions. After adding the solution of Alg with BP dropwise to AlCl_3_ solution, sphere-shaped gel formed abruptly due to electrostatic interaction amongst the carboxyl groups of Alg (negative charge) and Al^3+^ cations (positive charge). As a result, the carboxyl group of sodium alginate and Al^3+^ cations made it possible to cross-link with one another and convert the BP dispersed Alg solution into a gel that settled in AlCl_3_ aqueous solution in the form of beads.

**Scheme 1:**
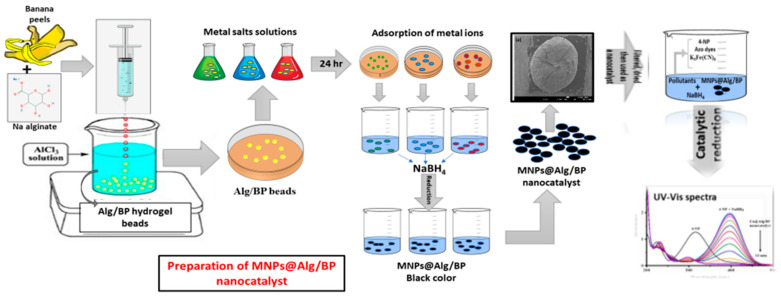
Graphical presentation of the preparation and application of the developed nanocatalysts.

A total of 0.2 g of Alg/BP beads were added to the metal salt solution (AgNO_3_, NiSO_4_·7H_2_O, Co(NO_3_)_2_·6H_2_O, FeCl_3_, CuSO_4_·5H_2_O) and kept overnight for adsorption of the metal ions. Beads acquired the color of the concerned salt solution after 24 h, which indicates the adsorption of the metal salt on the surface and interlayer of beads. The beads were removed from the salt solution and washed several times to eliminate the un-adsorbed metal. The metal adsorbed beads were further dipped in NaBH_4_ aqueous solution, and they turned black because NaBH_4_ reduced the adsorbed metal ions (Ag^+^, Ni^2+^, Co^2+^, Fe^3+^, and Cu^2+^) to the corresponding MNPs (AgNPs, NiNPs, CoNPs, FeNPs, and CuNPs). The preparation and applications of the developed catalysts are graphically shown in Scheme 1.

### 2.3. Instrumental Analysis

The morphology and the structure of MNPs@Alg/BP nanocatalysts were investigated using different analytical techniques. Scanning electron microscopy (FESEM from JEOL, JSM-7600F, Tokyo, Japan) was employed to study the morphology of MNPs@Alg/BP nanocatalysts. Energy dispersive X-ray spectroscopy (EDS) connected with FESEM was employed to analyze the composition of MNPs@Alg/BP nanocatalysts. X-ray diffraction (XRD) using a PANalytical diffractometer applying a Kα radiation (λ = 0.154 nm) source was employed to study the crystallinity of the sample. The functional groups of the MNPs@Alg/BP nanocatalysts were characterized by employing a Fourier transform infrared (ATR-FTIR) spectrometer (NicoletTM iS50 FTIR Spectrometer—Thermo Fisher Scientific, Waltham, MA, USA). ATR-FTIR was employed to record the spectrum in the range of 400 to 4000 cm^−1^. A UV-vis spectrophotometer was applied to examine the catalytic reduction of inorganic and organic contaminants in the presence of MNPs@Alg/BP nanocatalysts.

### 2.4. Catalytic Studies

MNPs@Alg/BP nanocatalysts were used to assess the catalytic reaction of inorganic and organic contaminants. The catalytic performance of MNPs@Alg/BP nanocatalysts was evaluated toward an inorganic pollutant K_3_[Fe(CN)_6_], a positively charged dye (MB), two negatively charged dyes (CR and MO) and 4-NP. K_3_[Fe(CN)_6_] was selected as an inorganic contaminant while the dyes and nitrophenol served as organic pollutants. The catalytic activity of MNPs@Alg/BP nanocatalysts was initially investigated in the catalytic reduction of K_3_[Fe(CN)_6_], 4-NP, CR, MO, and MB in the presence of NaBH_4_. A total of 2.5 mL of K_3_[Fe(CN)_6_] (conc. 2.0 mM), 4-NP (conc. 0.13 mM), CR (conc. 0.065 mM), MO (conc. 0.108 mM), and MB (conc. 0.065 mM) was initially placed in a quartz cuvette and UV spectrum was recorded using a UV-vis spectrophotometer. Then 0.5 mL of 0.1 M NaBH_4_ was added, and the UV spectrum was measured. Further, 5–15 mg of dried MNPs@Alg/BP nanocatalysts was added and UV-vis spectrum was continuously measured after every 1.0 min to monitor the catalytic reduction of the contaminant and the catalytic activity of MNPs@Alg/BP nanocatalysts. The reduction reactions were monitored by a decrease in λ_max_ of K_3_[Fe(CN)_6_] (415 nm), 4-NP (4-nitrophenolate ion = 415 nm), MO (460 nm), CR (490 nm), and MB (658 nm).

Equation (1) was applied to calculate the removal (%):

(1) $$\%R=\frac{C_{o}-C_{f}}{C_{o}}*100$$

where *C_o_* = initial concentration and *C_f_* = final concentration of contaminants.

## 3. Results and Discussion

### 3.1. Characterization

As previously mentioned, in order to overcome the agglomeration of Cu nanoparticles in the reaction mixture, they were applied to the surface of Alg@BP as a catalytic support, which effectively enhanced the performance of the nanocatalysts. Consequently, it is very important to characterize the structure of the prepared Cu@Alg/BP since the nature and structure of adsorbents play an essential role in catalytic reduction reactions of water-based pollutants for better aquatic purification. Accordingly, FESEM was employed to study the surface morphology and EDS was applied for elemental analysis of Cu@Alg/BP for further confirmation.

**Figure 1:**
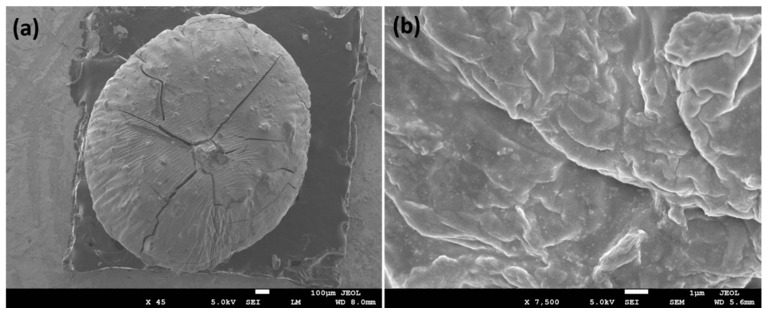
FESEM images of Cu@Alg/BP nanocatalyst at (**a**) low and (**b**) high magnifications.

In FESEM, the surface morphology was analyzed and the observed morphology of Cu@Alg/BP is shown in Figure 1. High-magnification FESEM shows a sphere shape bead of Alg/BP in the size range of 1.5–2.5 mm. As can be obviously seen in the low magnification FESEM image (Figure 1), spherical shaped particles were developed on the surface of the Alg/BP. Additionally, the surface of Alg/BP was coated with sufficiently separated metal nanoparticles. Obviously, the grooves seen on the surface were caused by the elimination of water, which resulted in shrinkage of the polymer matrix. The highly magnified image (Figure 1a) reveals the roughness of the surface with good dispersion indicating that Cu nanoparticles had been embedded in and on the surface of Alg/BP.

**Figure 2:**
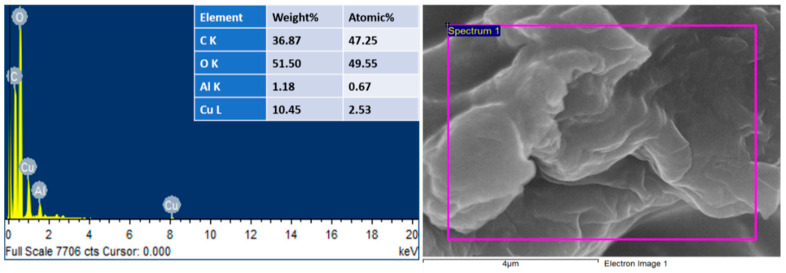
EDS of Cu@Alg/BP nanocatalyst.

EDS investigations also verified the successful preparation of Cu@Alg/BP. It showed peaks for carbon and oxygen as the main elements having 36.87 and 51.50 wt%, respectively, as shown in Figure 2. As previously indicated, banana peels contain organic compounds that are rich in carbon such as pectin substances, hemicellulose, and cellulose. It is also because of the several functional groups in the sodium alginate beads. Moreover, EDS spectra demonstrate a signal for Cu (10.45 wt%) suggesting the successful formation of such nanocomposites. The slight presence of Al (1.18 wt%) resulted from the crosslinker present in the sample.

### 3.2. Catalytic Properties

In order to evaluate the catalytic activity of MNPs@Alg/BP the catalytic reduction reactions of K_3_[Fe(CN)_6_], 4-NP, CR, MB, and MO were studied. Additionally, the MO reduction reaction was chosen for further investigation as it showed better results than other pollutants in terms of removal % and the reaction rate constant.

#### 3.2.1. Catalytic Reduction of 4-NP Using MNPs@Alg/BP

4-Aminophenol, which is formed from the reduction of 4-NP is commonly used to synthesize antipyretic and analgesic medicines such as paracetamol, phenacetin, and acetanilide. On the downside, 4-NP is naturally toxic and pollutes the biosphere when industries produce it. Therefore, its reduction is significantly needed by changing it to another nontoxic compound in the attempt to minimize its harmfulness. The reduction of 4-NP by NaBH_4_ using five different MNPs@Alg/BPs was investigated to evaluate the catalytic performance of the catalyst. Ag, Ni, Co, Fe, and Cu nanoparticles were incorporated with Alg/BP (1 bead).

**Figure 3:**
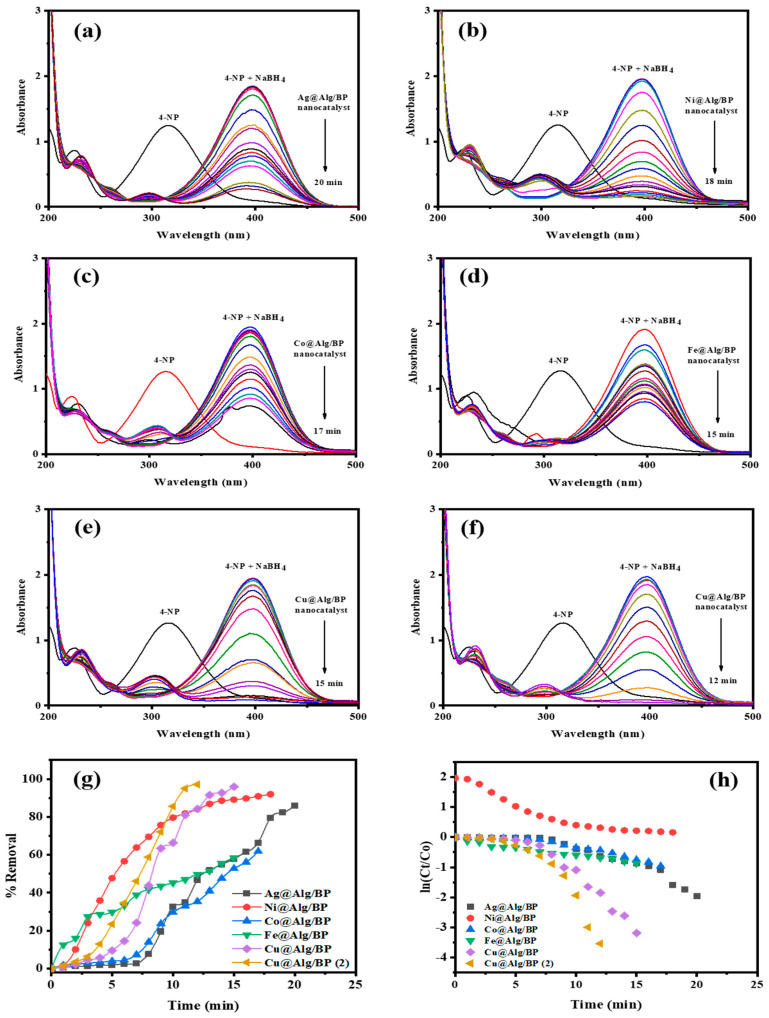
Time-dependent UV–vis spectra for 4-NP reduction by NaBH_4_ using 1 bead of (**a**) Ag@Alg/BP; (**b**) Ni@Alg/BP; (**c**) Co@Alg/BP; (**d**) Fe@Alg/BP; (**e**) Cu@Alg/BP; and 2 beads of (**f**) Cu@Alg/BP. (**g**) Removal % and (**h**) kinetics of pollutants reduction using nanocatalysts.

Noticeably, an absorption band at 400 nm appeared after the peak at 317 nm corresponding to 4-NP disappeared confirming that a nitrophenolate ion was formed, as shown in Figure 3. This shift to 400 nm can be attributed to the deprotonation of the –OH group existing in 4-NP. Visually, the only noticeable change during the reduction of 4-NP was the change of color from bright yellow to darker shade. Basically, this reaction is only thermodynamically feasible in an aqueous solution of BH_4_^-^. However, this reaction has a slow rate as a result of the large kinetic barrier caused by the large potential difference between the strong reducing agent (NaBH_4_) and 4-NP. As a result, a catalyst is required for 4-NP reduction because the energy barrier can be reached more quickly to complete the reaction at room temperature.

After addition of 10 mg of catalyst, the reduction process of 4-NP by the reducing agent was constantly evaluated using UV-visible spectroscopy where the changes in 4-NP concentration against time was recorded. A darker yellow color was observed when NaBH_4_ was added to the reduction reaction of 4-NP, which is naturally bright yellow, catalyzed by the MNPs@Alg/BP. A colorless solution was finally obtained within 20.0, 18.0 and 17.0 min, when Ag, Ni, and Co nanoparticles@Alg/BP (1 bead) were added, respectively. The complete reaction took only 15.0 min when Fe and Cu nanoparticles@Alg/BP were used. A shorter period of time (12.0 min) was achieved for competence of this catalytic reduction using Cu@Alg/BP (2 beads), as revealed in Figure 3f. A steady decrease was observed in the absorption peak at 400 nm. Meanwhile, an absorption peak at 300 nm was observed confirming the formation of the amino group of 4-AP for which the increase in absorption indicated that the reduction reaction was complete. The nanocatalyst (MNPs@Alg/BP) accelerated the transfer of electrons from BH_4_^–^ (the reducing agent) to 4-NP.

The removal % of 4-NP by the five different MNPs@Alg/BPs (1 bead) can be calculated using Equation (1). Values of 85.89%, 91.93%, 61.79%, 58.36% and 95.86% were obtained for the removal of 4-NP by converting it to 4-AP using Ag, Ni, Co, Fe, and Cu nanoparticles@Alg/BP, respectively, as shown in Figure 3g. The highest removal % (97.09%) was achieved by using Cu@Alg/BP (2 beads). Consequently, Cu@Alg/BP (2 beads) was further used for the other kind of pollutants owing to its efficiency in catalysis. The adsorption of 4-NP on the catalysts’ surfaces significantly influenced the step rate of the electron transference. 4-Nitrophenolate ions diffuse onto the nanocatalysts’ surfaces to react with the active proton species, which come from the adsorption of hydride ion (BH_4_^−^) on the nanocatalyst surface. The adsorbed nitro group reacts with BH_4_^−^ where the electrons are released from it to 4-NP resulting in the reduction of 4-NP to 4-AP.

The kinetics of MNPs@Alg/BP toward 4-NP reduction by NaBH_4_ follow a pseudo first-order rate [24], as shown in Equation (2):

ln *C_t_*/*C_o_* = ln *A_t_*/*A_o_* = −*Kt* (2)

where *C_t_* and *C_o_* are the concentrations of the pollutant and *A_t_* and *A_o_* are UV-Vis absorbances at designated time and t = 0, respectively. *K* (min^−1^) is the rate constant of the reduction reaction. Based on Equation (2), the slope of the plot of ln *C_t_*/*C_o_* versus reduction time (t) gives the value of *K_app_*, as presented in Figure 3h.

It was found that the values of *K_app_* for the catalytic reduction of 4-NP by NaBH_4_ using 1 bead of Ag@Alg/BP, Ni@Alg/BP, Co@Alg/BP, Fe@Alg/BP, and Cu@Alg/BP were 0.0930 min^−1^, 0.1043 min^−1^, 0.0564 min^−1^, 0.0517 min^−1^, and 0.2112 min^−1^, respectively. It was also revealed that the use of 2 beads of Cu@Alg/BP increased the rate constant to 0.2702 min^−1^.

#### 3.2.2. Reduction of K_3_[Fe(CN)_6_]

**Figure 4:**
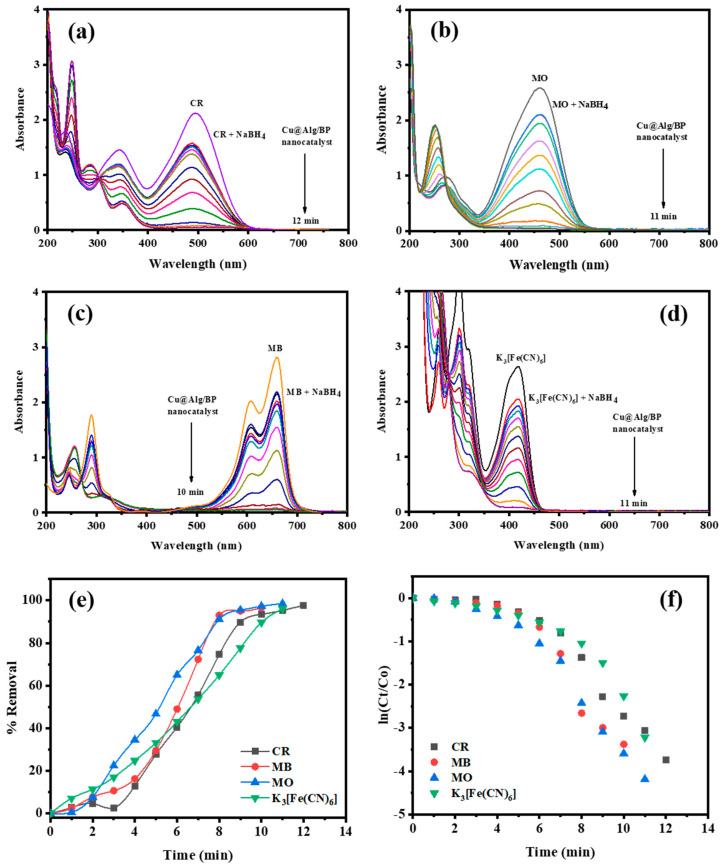
Time dependent UV–vis spectra for the reduction of (**a**) CR (0.065 mM); (**b**) MB (0.065 mM); (**c**) MO (0.108 mM); (**d**) K_3_[Fe(CN)_6_] (2.0 mM) by NaBH_4_ using 2 beads of Cu@Alg/BP. (**e**) Removal % and (**f**) kinetics of pollutants reduction using nanocatalysts.

The catalytic reduction reaction of K_3_[Fe(CN)_6_] as an inorganic pollutant was also studied using the prepared nanocatalysts (MNPs@Alg/BP). UV–vis absorption spectroscopy was effectively applied to monitor the kinetic reduction of this inorganic pollutant. First of all, a mixture of K_3_[Fe(CN)_6_] and NaBH_4_ was treated with 10 mg of Cu@Alg/BP (2 beads). The absorbance band at 415 nm gradually disappeared in 11.0 min due to the reduction of [Fe(CN)_6_]^3−^ to [Fe(CN)_6_]^4−^ (Fe^3+^ was reduced to Fe^2+^), as illustrated in Figure 4d. The catalytic reduction of this inorganic pollutant mainly relies on the process of the electron transfer. The reduction efficiency of Fe^3+^ to Fe^2+^ was evaluated by applying Equation (1). The results showed that 95.99% of the pollutant was removed whereas the K_app_ reached to 0.2472 min^−1^ (Figure 4e,f). This means that a successful conversion of K_3_[Fe(CN)_6_] was achieved by utilizing Cu@Alg/BP as an effective catalyst in presence of NaBH_4_.

According to the literature, this catalytic reduction process occurs in two steps. The polarization of the nanocomposite beads is involved in the first rapid step using NaBH_4_ (the reducing agent). In the following step, the excess surface electrons are transported to the contaminant of interest leading to formation of [Fe(CN)_6_]^4−^. Consequently, the reduction reaction is an electron-transfer process, as shown in the equation below:

BH_4_^−^ (aq)+ 8[Fe(CN)_6_]^3−^ (aq) + 3H_2_O (aq) → H_2_BO_3_^−^ (aq) + 8[Fe(CN)_6_]^4−^(aq) + 8H^+^

#### 3.2.3. Catalytic Reduction of Organic Dyes

##### Discoloration of CR Dye

Congo red dye is a benzidine-based anionic azo dye, known as [1-naphthalenesulfonic acid, 3,30-(4,40-biphenylenebi-s(azo)) bis(4-amino-) disodium salt], containing two azo groups in its chemical structure. This organic environmental toxicant is a carcinogenic dye that harmfully affects ecosystems. According to the literature, this dye is commonly applied to study the catalytic performance of several catalysts due to their dissociation in the presence of both catalyst and reducing agent. Similar to K_3_[Fe(CN)_6_], UV–vis spectroscopy was used to monitor the catalytic reduction of CR (0.065 mM) utilizing Cu@Alg/BP (2 beads) in the presence of NaBH_4_ in order to evaluate the effectiveness of the prepared nanocatalysts.

As the results revealed, the catalytic reduction of CR occurred in 12.0 min. The absorption band of this dye at 490 nm progressively disappeared. Meanwhile, a new absorbance peak at 290 nm appeared due to the complete catalytic reduction of CR by Cu@Alg/BP in the presence of the reducing agent. It has been reported that the azo (–N=N–) group in CR is reduced when the electrons from the reducing agent (BH_4_^-^) are moved to the nanocomposite beads before they are transferred to the dye (CR). In fact, the azo (–N=N–) double bond is catalytically reduced to a (–N–N–) single bond causing the disappearance of CR’s color. As previously mentioned, the conversion (%) was evaluated by applying Equation (1). The result showed that 97.61% of CR was reduced in 12.0 min by adding Cu@Alg/BP in presence of NaBH_4_ as revealed in Figure 4a,e. Based on Equation (2), the rate constant for CR reduction using Cu@Alg/BP (2 beads) was found to be 0.3184 min^−1^ (Figure 4f).

##### Discoloration of MO Dye

Similar to CR, MO is an organic pollutant that causes harmful impacts on aquatic life. The catalytic activity of Cu@Alg/BP (2 beads) in presence of NaBH_4_ was evaluated for a MO reduction using UV–vis spectroscopy. A new absorption peak at 250 nm appeared in 11.0 min while the first band at 460 nm gradually disappeared with the MO color confirming the completion of the catalyzed reduction of MO, as shown in Figure 4b. Similar to the CR reduction mechanism, the azo group (–N=N–) in MO is reduced to (–N–N–) when the electrons are transferred from the donor (BH_4_^-^) to the acceptor (MO) after their transmission to the catalyst (nanocomposite beads).

The conversion (%) and rate constant of MO reduction reaction by the nanocatalysts were also evaluated by applying Equations (1) and (2), respectively. The results showed that 98.48% of MO was reduced in 11.0 min by adding Cu@Alg/BP in presence of NaBH_4_, while the rate constant was 0.3975 min^−1^, as shown in Figure 4b–f.

##### Discoloration of MB Dye

Similar to other previous dyes, the same experiment was carried out to study the catalyzed reduction of MB by adding Cu@Alg/BP (2 beads) in the presence of NaBH_4_. As previously discussed, UV–vis spectroscopy was used to investigate the reduction reaction of MB in terms of the rate of reduction reaction and the effectiveness of the catalyst. The results proved that the completion of the reaction occurred in 10.0 min when the absorption band of MB at 658 nm completely disappeared. On the other hand, a new band at 240 nm appeared because of the reduction of MB, as clearly seen in Figure 4c. By applying Equations (1) and (2), the conversion % of MB was 96.58% in a shorter period of time and it had a high reaction rate up to 0.3574 min^−1^, as displayed in Figure 4e,f.

##### Possible Mechanism of Dyes Reduction

As previously discussed, the mechanism of the catalytic reduction of azo dyes (MO, CR, and MB) is based primarily on nanocomposite beads. At the beginning, the reducing agent (NaBH_4_) decomposes into an aqueous solution forming BH_4_^–^, which is considered a proton source. In the next step, the hydrogen produced from BH_4_^–^ attacks the dye’s molecules. Dye molecules then bind with MNPs@Alg/BP causing activation of azo bonds in dyes by the electrons carried by the nanocomposite beads. As a result of the bond conjugation, the azo bonds become weaker and then are broken down [25]. Double bond (–N=N–) is initially changed to single bond (–HN–NH–) which is then broken to form aromatic amines. Accordingly, the color of dyes disappear indicating the completion of the catalytic reduction. In other words, the catalyst works as an electron transferring material since it transfers the electron to the dye molecules (acceptor) from BH_4_^–^ (donor) resulting in an acceleration of the reduction reaction.

#### 3.2.4. Catalytic Reduction of MO

As previously described, MO was chosen as a model for further investigations because its catalytic reduction took place in a shorter time (11.0 min) and showed the highest removal % (98.48%) by adding Cu@Alg/BP (2 beads) in the presence of NaBH_4_. Accordingly, the impact of various parameters on the catalytic reduction reaction of pollutants by MNPs@Alg/BP with the reducing agent (NaBH_4_) was further studied. The performance of the prepared Cu@Alg/BP was also compared with other catalysts reported in the literature for catalyzed reduction of MO in the presence of NaBH_4_, as revealed in Table 1 [25,26,27,28,29,30].

##### Effect of Different MNPs@Alg/BP on MO Reduction

**Figure 5:**
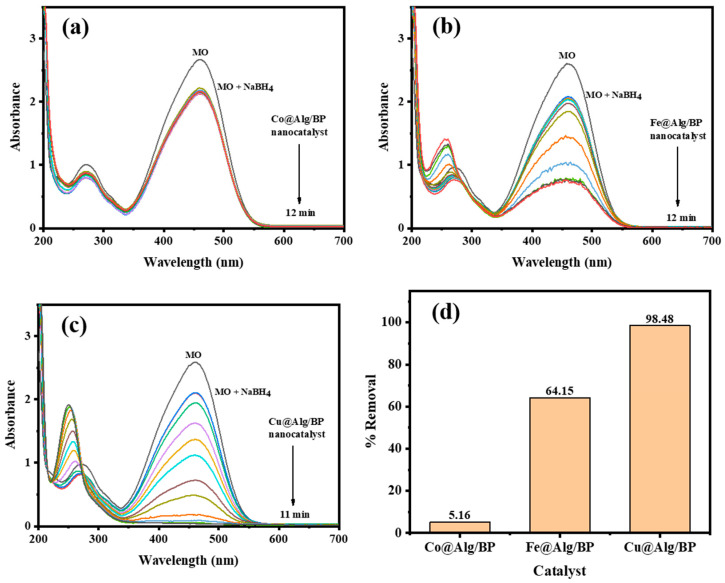
Time-dependent UV–vis spectra for MO reduction by NaBH_4_ using 2 beads of (**a**) Co@Alg/BP; (**b**) Fe@Alg/BP; and (**c**) Cu@Alg/BP nanocatalysts. (**d**) Removal % of MO reduction using nanocatalysts.

In order to examine the influence of using different metal nanoparticles with Alg/BP (2 beads) on the catalytic reduction of MO by NaBH_4_, Co@Alg/BP and Fe@Alg/BP were selected to be added to the reaction. As previously discussed, Co and Fe showed relatively poor removal % (61.79% and 58.36%), respectively, when they were incorporated in the catalyst for the reduction of 4-NP, as illustrated in Figure 3c,d. Similarly, the reduction of MO in terms of the rate of reduction reaction and the effectiveness of the catalyst were investigated by employing UV–vis spectroscopy. The findings confirmed that the completion of MO reduction occurred in 12.0 min for both with different removal %. By applying Equation (1), the conversion % of MO by adding Fe@Alg/BP was 64.15% in 12.0 min whereas only 5.16% was achieved for the same duration of time when Co@Alg/BP was used, as shown in Figure 5a,b. On the other hand, superior removal % (98.48%) was achieved in 11.0 min when Cu@Alg/BP was added to the MO reduction reaction, as mentioned above and is shown in Figure 5c,d. To summarize, it was confirmed by the results that Cu@Alg/BP is the most effective nanocatalyst in terms of proficiency and the reaction rate of MO reduction.

##### Effect of Catalyst Amount on MO Reduction

**Figure 6:**
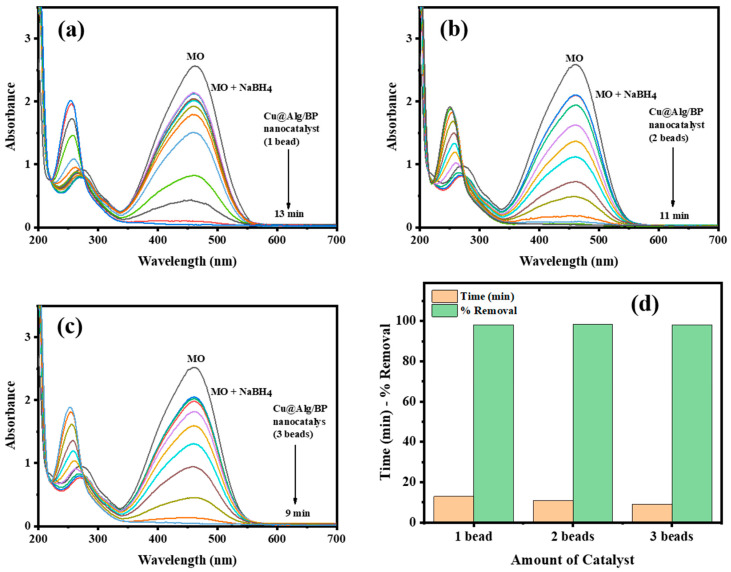
Time-dependent UV–vis spectra for MO reduction by NaBH_4_ using (**a**) 1 bead; (**b**) 2 beads; (**c**) 3 beads of Cu@Alg/BP nanocatalyst. (**d**) Comparative relation of % removal and time required for MO reduction using nanocatalysts.

In order to examine the impact of quantity of Cu@Alg/BP on MO reduction, three amounts of catalyst (1, 2 and 3 beads) were used. The other parameters were kept unchanged to investigate only the effect of the catalyst dosage on the reaction. Similarly, the reduction of MO was examined using UV–vis spectroscopy in terms of the rate of reduction reaction and the effectiveness of the catalyst. The results demonstrated that removal % of MO remained unchanged by altering the catalyst dosage. In contrast, the reaction rate of MO reduction was slightly influenced. There is inverse proportion between the reaction rate and the catalyst amount. The reduction of MO took place in 13.0 min when the smallest amount of catalyst (1 bead) was used with the reducing agent, as shown in Figure 6a. On the other hand, it needed 9.0 min to reach the completion when the greatest quantity of catalyst (3 beads) was added, as illustrated in Figure 6c. As previously mentioned, the reduction finished in 11.0 min if 2 beads of catalyst were used, as seen in Figure 6b. In parallel to the above results, 4-NP was also reduced by NaBH_4_ in the presence of 1 bead and 2 beads of Cu@Alg/BP and similar results were obtained for reaction rate and time duration. The reaction took a shorter time (12.0 min) in the presence of 1 bead of catalyst and a longer time (15.0 min) with 2 beads. In conclusion, the rate of catalytic reaction of dye is inversely proportional with the amount of catalyst used to accelerate it in the presence of NaBH_4_, as clearly illustrated in Figure 6d.

##### Effect of MO Concentration on Reduction Reaction Rate

**Figure 7:**
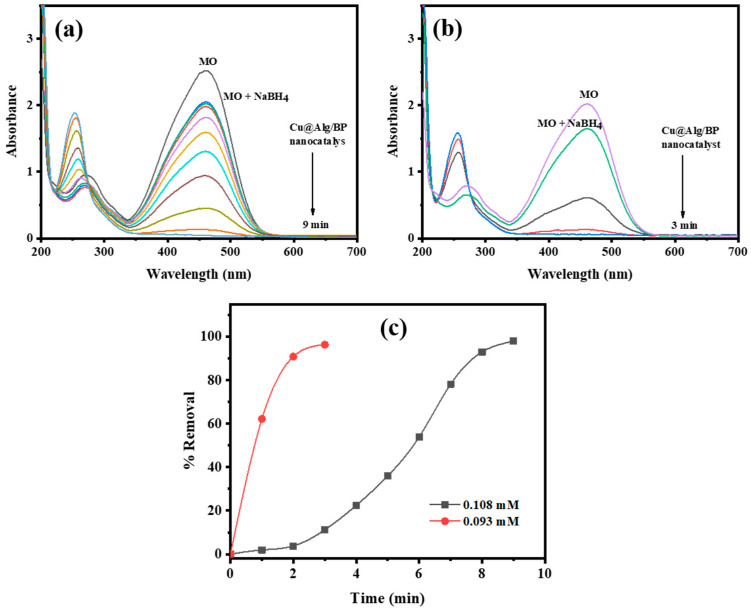
Time-dependent UV–vis spectra for MO reduction (**a**) 0.108 mM; (**b**) 0.093 mM by NaBH_4_ using 3 beads of Cu@Alg/BP nanocatalyst. (**c**) Removal % of MO in presence of Cu@Alg/BP.

Two different MO concentrations (0.108 and 0.093 mM) were used to study the impact of MO concentration on the duration of time the reduction reaction takes to finish in the presence of NaBH_4_ and Cu@Alg/BP (3 beads). The amount of catalyst was selected to be 3 beads since it previously gave the shortest time for reaction completion as mentioned above. The other reaction parameters were kept unchanged in order to study the effect of MO concentration on the rate of its reduction. Similarly, UV–vis spectroscopy was used to monitor the reduction of the two MO concentrations. It was confirmed by obtained data that the rate of the reduction reaction was noticeably increased by approximately 33% with a lower concentration (0.093 mM), as shown in Figure 7a,b. The reduction finished in 9.0 min when 0.108 mM of MO was added in presence of NaBH_4_ and the nanocatalyst, while it took only 3.0 min with a more diluted MO (0.093 mM) under the same reaction conditions. On contrary, the removal % of MO was approximately the same in both cases; however, the dye was reduced slightly more when a greater concentration of MO was used, as shown in Figure 7c. In conclusion, the concentration of MO is directly proportional to the duration of time it takes the catalytic reduction reaction to finish and has a slight positive impact on the conversion %.

##### Effect of NaBH_4_ Concentration on MO Reduction

**Figure 8:**
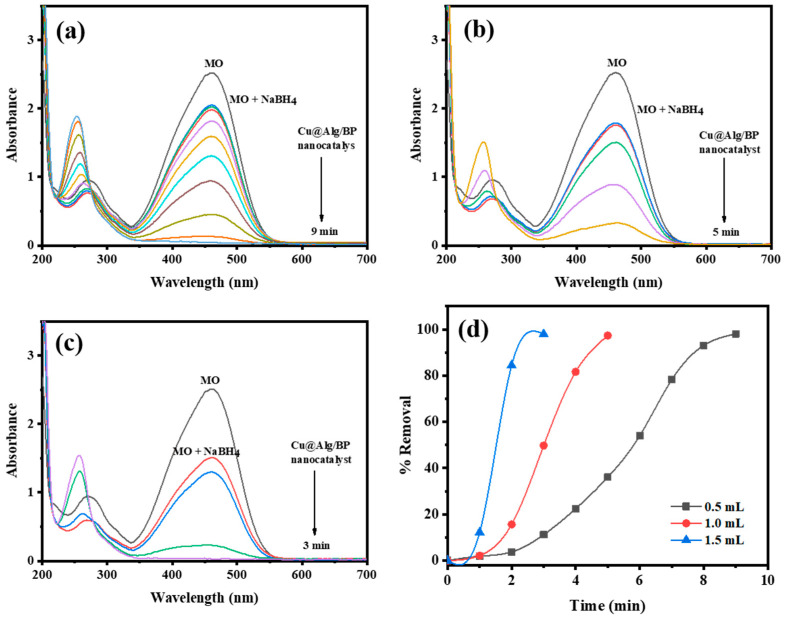
Time-dependent UV–Vis spectra for reduction of MO by different concentrations of NaBH_4_ (**a**) 0.5 mL; (**b**) 1.0 mL; and (**c**) 1.5 mL using 3 beads of Cu@Alg/BP nanocatalyst. (**d**) Removal % of MO in presence of Cu@Alg/BP.

Similarly, different concentrations of reducing agent (NaBH_4_) (0.5, 1.0, and 1.5 mL) were used to study the impact of the concentration of NaBH_4_ on the reduction of MO by adding 3 beads of Cu@Alg/BP. The amount was selected to be 3 beads since it increased the reduction rate as previously confirmed. The other reaction parameters were kept unchanged in order to study the influence of NaBH_4_ amount on the catalyzed reduction of MO. Similar to previous experiments, UV–Vis spectroscopy was used to monitor the reduction of MO by adding 3 different concentrations of NaBH_4_. The results showed that the removal % of MO was unaffected by changing the concentration of the reducing agent, as obviously noted in Figure 8d. In contrast, the reduction rate was remarkably influenced by altering the concentration. The reduction of the dye was faster by increasing the concentration of NaBH_4_, as shown in Figure 8a–c. In other words, the amount of reducing agent positively influences the catalytic reduction of MO. The reduction occurred in only 3.0 min when 1.5 mL of reducing agent was added. Smaller quantities (0.5 and 1.0 mL) of NaBH_4_ influenced the duration of the time the reduction takes to 9 and 5.0 min, respectively. Accordingly, the reduction rate of MO is directly proportional with the concentration of the reducing agent. As mentioned earlier, the electrons are transferred from the donor (reducing agent) to the acceptor (dye) through the nanocatalyst. Therefore, when the concentration of a species that is rich in electrons is increased, it rapidly circulates in the reaction media resulting in fast electron transference to the dye by the nanocatalyst.

##### Recyclability of Cu@Alg/BP

**Figure 9:**
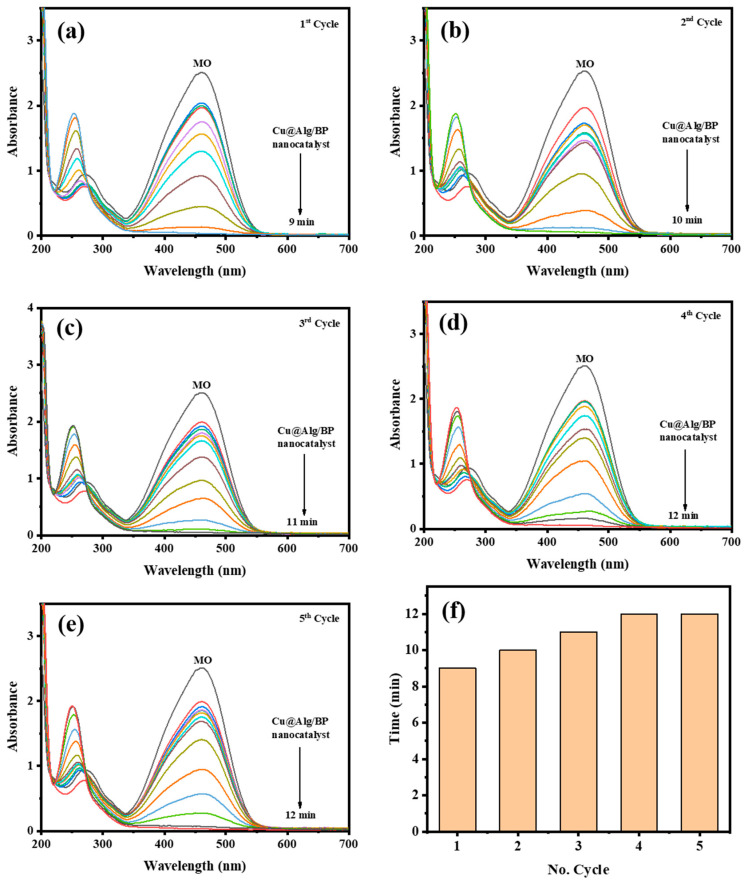
(**a**–**e**) Time-dependent UV–Vis spectra for MO reduction by NaBH_4_ using 3 beads of Cu@Alg/BP nanocatalyst five times. (**f**) Recyclability of Cu@Alg/BP nanocatalyst for MO reduction.

Sufficient recovery and stability of any catalyst are vital due to its cost-value and eco-friendliness. Hence, the recyclability of Cu@Alg/BP (3 beads) used for MO catalytic reduction was investigated. The recovery of Cu@Alg/BP nanocatalyst was tested four times consecutively. After each test, the nanocatalyst beads were simply removed from the product and washed then with distilled water. The results proved the stability of the catalyst since it could be recycled several times with no impact on its catalytic performance. The time that MO catalytic reduction took in each cycle using Cu@Alg/BP nanocatalyst was evaluated, as shown in Figure 9. The results demonstrated that Cu@Alg/BP nanocatalyst could reduce MO in 12.0 min until the 5th cycle confirming its stability and recyclability. These findings suggest that Cu@Alg/BP nanocatalysts can be used for several cycles with no significant impact on catalytic activity.

##### Application to Real Samples

**Figure 10:**
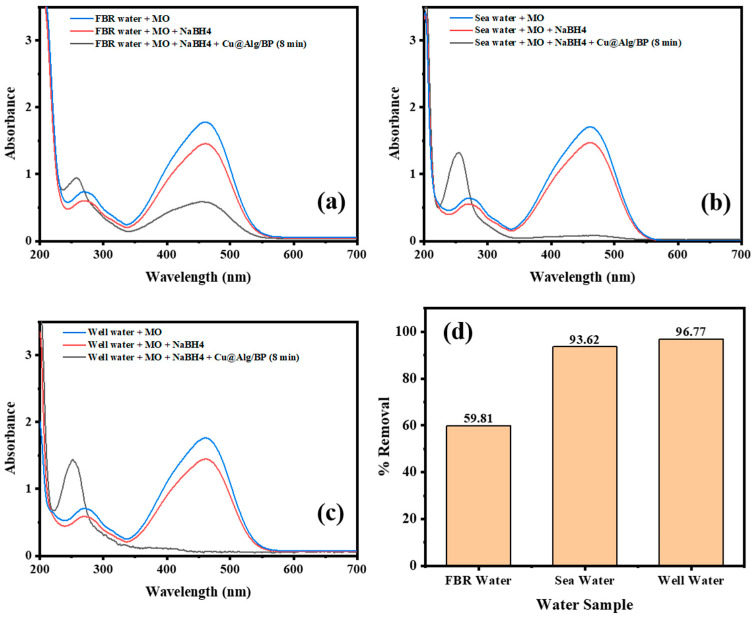
Time-dependent UV–Vis spectra for MO reduction by NaBH_4_ using 3 beads of Cu@Alg/BP nanocatalyst in (**a**) FBR water; (**b**) sea water; and (**c**) well water. (**d**) Catalytic reduction of MO in real samples after 8.0 min contact time.

After optimization of the catalytic reduction process, three real samples were used, seawater, well water, and agricultural wastewater (FBR), to study the conversion % of the pollutant (MO) by adding Cu@Alg/BP (3 beads) with the use of NaBH_4_. Similar to the experiments described above, the catalytic reduction of MO was investigated using a UV–Vis spectrophotometer. All real sample solutions were prepared by adding 2 mL of MO solution to 1 mL of real sample. A total of 0.5 mL of NaBH_4_ was then added to the solution in the UV cuvettes. Three beads of the nanocatalyst were added to each sample to accelerate the reduction of MO. In order to examine the removal % of MO in all water samples, the absorbance of the solution containing only MO with the water sample was measured. NaBH_4_ was then added to the solution and the absorbance was measured again. Finally, Cu@Alg/BP (3 beads) was added to the previous solution in order to accelerate the reduction of MO. The contact time was set to 8.0 min for all water samples. The results showed that the % reduction of MO was significantly different in case of FBR sample compared with the other two samples. It was 59.81% in FBR which is much lower than the other two samples, which achieved more than 90%, as illustrated in Figure 10. Indeed, MO was effectively removed by 96.77% and 93.62% in well water and seawater samples, respectively, as shown in Figure 10b,c. In other words, the pollutant (MO) in the sample of well water was mostly converted to a safer product causing the disappearance of its absorption peak at 460 nm. Noticeably, this excellent result, achieved in 8.0 min, is in agreement with the result obtained previously (9.0 min) for MO reduction when 0.5 mL of NaBH_4_ and 3 beads of Cu@Alg/BP were added, as shown above in Figure 8a. In contrast, MO was partially removed from the FBR water sample and its absorbance band at 460 nm did not disappear completely, as revealed in Figure 10a. The seawater sample showed excellent removal of MO; however, it is slightly lower than the well water sample, which has less interferences. Overall, the catalytic reduction of MO in well- and seawater samples by adding Cu@Alg/BP is a successful, robust, and feasible technique that can be used to remove pollutant (MO).

## 4. Conclusions

This study depicted the catalytic reduction of many different environmental pollutants, K_3_[Fe(CN)_6_], 4-NP and three different dyes (MO, CR and MB), based on MNPs@Alg/BP catalysts in presence of NaBH_4_. This effective alginate/banana waste-based composite was successfully prepared using a robust and feasible eco-friendly method. Banana peels were efficiently converted into a useful material that could be later employed for remedying water from organic and inorganic pollutants. It was found that among many different nanocatalysts, Cu@Alg/BP was the most effective catalyst in terms of efficiency, reduction rate, and removal % of the pollutants (4-NP and MO). As data revealed, the rate of catalytic reduction (MO) is inversely proportional with the amount of catalyst used for enhancement and no impact of this catalyst dosage on removal % of pollutant (MO). It was also confirmed that the concentration of the toxicant (MO) is directly proportional to the time duration the catalytic reduction takes to finish and has a slight positive influence on the conversion % of pollutant. In addition, the results proved that the removal % of toxicant (MO) is unaffected by changing the concentration of the reducing agent whereas the reduction rate is directly proportional with NaBH4 concentration. Real sample analyses confirmed the efficacy of MNPs@Alg/BP for the removal of water contaminants. It can be concluded that a new effective strategy for the removal of inorganic and organic water contaminants was successfully created to be used as an alternative to high-cost commercial catalysts.

## Figures and Tables

**Table 1:** Comparison of rate constant values of MO reduction reactions measured for different catalysts, reported in the literature, in presence of NaBH_4_.

Catalyst	Rate Constant (min^−1^)	Reference
K-AgNPs	0.0286	[25]
CuO nanosheets	0.0069	[26]
CuSnOS/AC	1.1480	[27]
AuNP- Myristica fragrans	0.5136	[28]
PEP-AgNPs	0.3588	[29]
PEP-AuNPs	0.2556	[29]
Pd NPs	0.0909	[30]
Cu@Alg/BP	0.2702	Current Study

## Data Availability

Not applicable.

## References

[1] Kamal T., Ahmad I., Khan S.B., Asiri A.M. (2019). Anionic polysaccharide stabilized nickel nanoparticles-coated bacterial cellulose as a highly efficient dip-catalyst for pollutants reduction. React. Funct. Polym..

[2] Khan M.S.J., Kamal T., Ali F., Asiri A.M., Khan S.B. (2019). Chitosan-coated polyurethane sponge supported metal nanoparticles for catalytic reduction of organic pollutants. Int. J. Biol. Macromol..

[3] Zhu Y., Fan W., Zhou T., Li X. (2019). Removal of chelated heavy metals from aqueous solution: A review of current methods and mechanisms. Sci. Total Environ..

[4] Ahmad M.A., Eusoff M.A., Oladoye P.O., Adegoke K.A., Bello O.S. (2012). Optimization and batch studies on adsorption of Methylene blue dye using pomegranate fruit peel based adsorbent. Chem. Data Collect..

[5] Pham T.H., Jung S.H., Kim Y.J., Kim T. (2021). Adsorptive removal and recovery of organic pollutants from wastewater using waste paper-derived carbon-based aerogel. Chemosphere.

[6] Kurniawan T.A., Chan G.Y.S., Lo W.-H., Babel S. (2006). Comparisons of low-cost adsorbents for treating wastewaters laden with heavy metals. Sci. Total Environ..

[7] Zhou Y., Lu J., Zhou Y., Liu Y. (2019). Recent advances for dyes removal using novel adsorbents: A review. Environ. Pollut..

[8] Nayak S.S., Mirgane N.A., Shivankar V.S., Pathade K.B., Wadhawa G.C. (2021). Adsorption of methylene blue dye over activated charcoal from the fruit peel of plant hydnocarpus pentandra. Mater. Today Proc..

[9] Ahmad T., Danish M. (2018). Prospects of banana waste utilization in wastewater treatment: A review. J. Environ. Manag..

[10] Farirzadeh I., Samani M.R., Toghraie D. (2020). Lead removal from aqueous medium using fruit peels and polyaniline composites in aqueous and non-aqueous solvents in the presence of polyethylene glycol. Chin. J. Chem. Eng..

[11] Mahindrakar K.V., Rathod V.K. (2018). Utilization of banana peels for removal of strontium (II) from water. Environ. Technol. Innov..

[12] Hashem A.H., Saied E., Hasanin M.S. (2020). Green and ecofriendly bio-removal of methylene blue dye from aqueous solution using biologically activated banana peel waste. Sustain. Chem. Pharm..

[13] Solangi N.H., Kumar J., Mazari S.A., Ahmed S., Fatima N., Mubarak N.M. (2021). Development of fruit waste derived bio-adsorbents for wastewater treatment: A review. J. Hazard. Mater..

[14] Yu D., Wang L., Wu M. (2018). Simultaneous removal of dye and heavy metal by banana peels derived hierarchically porous carbons. J. Taiwan Inst. Chem. Eng..

[15] Ali A., Saeed K., Mabood F. (2016). Removal of chromium (VI) from aqueous medium using chemically modified banana peels as efficient low-cost adsorbent. Alex. Eng. J..

[16] Munagapati V.S., Yarramuthi V., Kim Y., Lee K.M., Kim D.-S. (2018). Removal of anionic dyes (Reactive Black 5 and Congo Red) from aqueous solutions using Banana Peel Powder as an adsorbent. Ecotoxicol. Environ. Saf..

[17] Zhou Y., Jin C., Li Y., Shen W. (2018). Dynamic behavior of metal nanoparticles for catalysis. Nano Today.

[18] Jang E.S., Khan S.B., Seo J., Nam Y.H., Choi W.J., Akhtar K., Han H. (2011). Synthesis and characterization of novel UV-curable polyurethane–clay nanohybrid: Influence of organically modified layered silicates on the properties of polyurethane. Prog. Org. Coat..

[19] Lee Y., Kim D., Seo J., Han H., Khan S.B. (2013). Preparation and characterization of poly(propylene carbonate)/exfoliated graphite nanocomposite films with improved thermal stability, mechanical properties and barrier properties. Polym. Int..

[20] Gul S., Rehan Z.A., Khan S.A., Akhtar K., Khan M.A., Khan M.I., Rashid M.I., Asiri A.M., Khan S.B. (2017). Antibacterial PES-CA-Ag_2_O nanocomposite supported Cu nanoparticles membrane toward ultrafiltration, BSA rejection and reduction of nitrophenol. J. Mol. Liq..

[21] Sachan N., Pushkar S., Jha A., Bhattcharya A. (2009). Sodium alginate: The wonder polymer for controlled drug delivery. J. Pharm. Res..

[22] Xia M., Kang S.-M., Lee G.-W., Huh Y.S., Park B.J. (2019). The recyclability of alginate hydrogel particles used as a palladium catalyst support. J. Ind. Eng. Chem..

[23] Khan S.B., Ahmad S., Kamal T., Asiri A.M., Bakhsh E.M. (2020). Metal nanoparticles decorated sodium alginate-carbon nitride composite beads as effective catalyst for the reduction of organic pollutants. Int. J. Biol. Macromol..

[24] Khan F.U., Asimullah, Khan S.B., Kamal T., Asiri A.M., Khan I.U., Akhtar K. (2017). Novel combination of zero-valent Cu and Ag nanoparticles @ cellulose acetate nanocomposite for the reduction of 4-nitro phenol. Int. J. Biol. Macromol..

[25] Sherin L., Sohail A., Amjad U.-e.-S., Mustafa M., Jabeen R., Ul-Hamid A. (2020). Facile green synthesis of silver nanoparticles using Terminalia bellerica kernel extract for catalytic reduction of anthropogenic water pollutants. Colloid Interface Sci. Commun..

[26] Sahu K., Singh J., Mohapatra S. (2019). Catalytic reduction of 4-nitrophenol and photocatalytic degradation of organic pollutants in water by copper oxide nanosheets. Opt. Mater..

[27] Sun H., Abdeta A.B., Kuo D.-H., Wu Q., Guo Y., Zelekew O.A., Yuan Z., Lin J., Chen X. (2021). Activated carbon supported CuSnOS catalyst with an efficient catalytic reduction of pollutants under dark condition. J. Mol. Liq..

[28] Punnoose M.S., Bijimol D., Mathew B. (2021). Microwave assisted green synthesis of gold nanoparticles for catalytic degradation of environmental pollutants. Environ. Nanotechnol. Monit. Manag..

[29] My-Thao Nguyen T., Anh-Thu Nguyen T., Tuong-Van Pham N., Ly Q.-V., Thuy-Quynh Tran T., Thach T.-D., Nguyen C.-L., Banh K.-S., Le V.-D., Nguyen L.-P. (2021). Biosynthesis of metallic nanoparticles from waste Passiflora edulis peels for their antibacterial effect and catalytic activity. Arab. J. Chem..

[30] Narasaiah B.P., Mandal B.K. (2020). Remediation of azo-dyes based toxicity by agro-waste cotton boll peels mediated palladium nanoparticles. J. Saudi Chem. Soc..

